# Monthly Report of Diseases

**Published:** 1801-02

**Authors:** 


### MONTHLY REPORT of DISEASES,

Admitted under the Care of the PHYSICIANS of the FINSBURY DISPENSARY, St. John's Square, Clerkenwell.

From Dec. 20, 1800, to Jan. 20, 1801.

| | No. of Caſes. | | No. of Caſes. |
|---|---|---|---|
| Continued Fever - - | 69 | Menorrhagia - - - - - | 7 |
| Small-Pox - - - - - - | 3 | Melancholia - - - - - | 1 |
| Eryſipelas - - - - - - | 5 | Dyſpepſia - - - - - - | 16 |
| Cynanche Tonſillarum - | 7 | Hyſteria - - - - - - | 3 |
| Phthiſis Pulmonalis - - | 11 | Paralyſis - - - - - - | 4 |
| Hæmoptyſis - - - - | 4 | Gaſtrodynia and Enterodynia | 6 |
| Pneumonia - - - - - | 2 | Epilepſia - - - - - - | 4 |
| Cough and Dyſpnœa - - | 57 | Cephalæa - - - - - - | 7 |
| Catarrh - - - - - - - | 4 | Dropſy - - - - - - - | 11 |
| Dyſentery - - - - - - | 2 | Aſthenia - - - - - - | 19 |
| Diarrhœa - - - - - - | 6 | Infantile Diſeaſes - - - | 13 |
| Chloroſis and Amenorrhœa | 18 | Chronic Eruptions - - | 11 |

The caſes of typhus will appear to have very conſiderably increaſed in number during the laſt month; but they have not, in general, been characteriſed by that violence of frenzy which was ſo eſpecially remarkable in the fevers of the ſummer and autumn. The extraordinary determination to the head that prevailed during thoſe ſeaſons, in various inſtances, produced not merely the common delirium of fever, but an abſolute and a ſpeedily fatal inſanity.

The preſent typhus is more particularly marked by a profound coma, and a general proſtration of the ſtrength.

In ſeveral caſes that at firſt ſeemed to be deſperate, a recovery, it might almoſt be called a reſurrection, was brought about principally by very ſtrong ſtimuli, applied in ſmall but frequently repeated doſes. After the inſide had been *rinced* by emetics and aperients, brandy with wine was often adminiſtered in as large a quantity as the patient was ſuppoſed able to ſwallow, without the inconvenience of ſucceeding ſickneſs or intoxication.

This mode of treatment, when it was aſſiſted by the daily waſhing of the whole body with cold water, the free admiſſion of freſh air into the ſick room, the frequent change of linen and bed clothes, moderate doſes of opium at night, and the careful abſtraction of every thing that might tend to interrupt the ſleep of the patient, or to diſturb the tranquillity of his mind, has ſeldom failed to induce a ſpeedy convaleſcence, the progreſs of which towards an entire reſtoration of health and ſtrength was afterwards accelerated by the application of nouriſhing diet, and a variety of medicinal corroborants.

It ſhould, however, be underſtood, that the directions of the phyſician, thoſe eſpecially that related to the adminiſtration of wine and food, could not, in many caſes, be carried into effect, in conſequence of the poverty of the patient, and the ſlow and ſcanty aſſiſtance of parochial charity.

Too generally is it imagined that work-houses are not provided only, but also are fully adequate to the relief of those who labour under the extremity of want. But if any credit may be paid to the testimonies of many of the poor creatures that crawl to supplicate the medical relief of the Dispensary, this is far from being, in every instance, actually the case.

Not longer since than yesterday, to the person who is now employed in writing this Report, a boy was brought, whose attenuated and nearly lifeless form awakened a sentiment, and even an exclamation of compassion from all the surrounding patients.

According to the mother's account, whose appearance and manner gave not the slightest pretence for suspecting the veracity of her assertions, her child had been for some time deposited in a work-house, but had there been deprived of what was absolutely essential not to his health merely but almost to the bare support of his existence.

By some the Reporter has been charged with indiscretion, in thus unveiling the extreme wretchedness of the poor. But, deaf to the dictates of worldly prudence, and insensible to the impulses of selfish interest, never, by such motives, will he be tempted to decline the declaration of facts which he knows to be true, and the discovery of which he cannot but regard as of unspeakable importance.*

One of the cases of melancholia, recorded in the list of diseases, is deserving of attention, from the circumstance of the patient's not having been afflicted with it until after the deprivation of his sight. The reflection upon that loss could not fail for a time to have been itself a course of uneasy feelings; but the continuance of his depression might perhaps be better accounted for by his not being any longer able, in consequence of this loss, to pursue his usual active employment, by its withdrawing from him the natural and exhilarating stimulus of light, and by its precluding altogether the possibility of that amusement and diversion of mind, which in general is so constantly derived from the contemplation of external objects. By confining the sensibility within a smaller compass, it condensed and increased its force.

A case occurred which the physician who was consulted with regard to it, suspected to be an aneurism of the aorta. But from a degree of diffidence with regard to the accuracy of his conclusion, he had recourse, for a removal of his doubts, to the advice of a respectable surgeon†, who, after an attentive examination, having formed

* Since the publication of the last Report, a donation of twenty pounds has been presented, by a person well known for his humanity toward the poor, to the physicians of the Finsbury, and several other Dispensaries in London, for the relief of those of their necessitous patients who are confined to a sick bed; and, to use the words of the gentleman through whose medium it was transmitted, "to be distributed in small sums proportioned to *the number of starving children*."

† If the name of the gentleman alluded to was to be mentioned, *it would not only* give an additional confirmation to the fact, but also would demonstrate to the public the extreme uncommonness of a case that could have been mistaken by a person of his professional learning and sagacity.

formed more decidedly the fame opinion, fcrupled not inftantly to pronounce the fentence of fpeedy death upon the unfortunate patient.

Since that time, however, there has occurred ftrong reafon to believe that the c. mplaint was merely a nervous affection. It appears to have originated from diftrefs of mind; it was induced often, and always was aggravated by any thing that agitated her fpirits; it was accompanied by Borboriemi; all thefe fymptoms uniformly increafed or diminifhed together: For about ten weeks they have all been gradually difappearing; and for this laft fortnight have fcarcely been felt at all, in confequence, as it is reafonable to fuppofe, of the remembrance of the painful circumftances from which her diforder had at firft originated, having, during that time, become more feeble; and alfo perhaps from a favourable change that, according to her own account, had lately taken place in the ftate of her external circumftances.

In many difeafes, efpecially in thofe that are called nervous, a fufpenfion of the fymptoms has not unfrequently been found to occur in confequence of the fame, or indeed any other complaint attacking one who is particularly near and interefting to the patient. Even in cafes of fever, a mother feldom finds herfelf fick until the ficknefs of her child is over. The attention and anxiety which are conftantly kept up, do not prevent the body from receiving contagion, but often prevent the mind from being for fome time aware of its influence. No fooner, however, does the recovery of the child take place, than, in general, it is fucceeded by the indifpofition of the parent; which, although it has been delayed, cannot fail to be aggravated and rendered ftill more dangerous by the extreme degree of labour, watchfulnefs, and folicitude that had immediately preceded its attack.

In the remarks which, in a former part of this report, have been made with regard to the ufe of ftimuli in cafes of defperate difeafe, it was far from the intention of the writer to encourage a recourfe to them in a ftate of health and vigour. The application of any extraordinary ftimulus to the human fyftem muft invariably abridge the period of its poffible duration; but at the moment when it is about to be extinguifhed, it is neceffary to *blow* the flame of life, although by that means you confume a part of the fuel that is neceffary to its fupport.

The grand object to be attended to in what is vulgarly denominated a low fever, is by artificial means to give, for a time, a degree of ftrength that may fupport the patient during *the period of his ftruggle with death.*

The ftimulating mode of practice feems to be particularly fuited to the conftitution of thofe who live under the influence of the fedative atmofphere of the metropolis. In London it is fcarcely poffible for a man to receive into his lungs a draught of air that has not been in fome other perfon's lungs before. This *second hand* air, is not only always injurious to health, but fometimes proves almoft immediately deftructive of exiftence.

It

It is a circumſtance well worthy of remark, that out of the multitude of caſes that within the extent of the Finſbury diſtrict have, for this laſt year, been attacked by typhoid contagion, few, comparatively, have fallen victims to it, but thoſe whoſe previous habits of debauchery or intemperance had reduced them into a ſtate that was peculiarly ſuſceptible of diſeaſe, and almoſt altogether inſenſible to the operation of medicine. Bark has but a feeble effect upon a ſtomach that has been long accuſtomed to brandy; and a frame, the ſtamina of which have been deſtroyed by an inordinate indulgence of the ſenſual appetites will, in no inſtance, be regenerated, or even find conſiderable relief, from a recourſe to any of the pharmaceutical preparations*.

*Red-lion Square.* J. R.†

* The idea which it is here meant to inculcate, and indeed has been more than once inculcated in theſe reports, has ſeldom been enough attended to by patients, or acted upon in the practice of phyſicians. Upon the minds of the former its truth cannot too deeply be impreſſed, although from the future profits of the latter a conviction of its truth might chance to deduct no ſmall portion of that pecuniary emolument which they have hitherto been in the habit of extracting from the mental, more perhaps than from the corporeal imbecility of their fellow creatures.

† Dr. Webb having retired from his official connection with the Finſbury Diſpenſary, theſe reports cannot any more be ſanctioned by the initials of a name that long will be remembered; and as long as it is remembered, will be reſpected by the ſupporters, and ever will be dear to the memory of the patients of that charitable inſtitution. From the friendſhip and ſociety of his late amiable colleague, the writer of this article has been indebted for relief under the preſſure of almoſt unprecedented calamity; and from his kindneſs and profeſſional ſkill has derived important aſſiſtance in diſcharging the duties annexed to an anxious and moſt laborious ſituation.

